# Barriers to Diffusion in Dendrites and Estimation of Calcium Spread Following Synaptic Inputs

**DOI:** 10.1371/journal.pcbi.1002182

**Published:** 2011-10-13

**Authors:** Armin Biess, Eduard Korkotian, David Holcman

**Affiliations:** 1 Bernstein Center for Computational Neuroscience, Göttingen, Germany; 2 Max-Planck-Institute for Dynamics and Self-Organization, Göttingen, Germany; 3 Department of Neurobiology, Weizmann Institute of Science, Rehovot, Israel; 4 Department of Computational Biology, Ecole Normale Supérieure, Paris, France; University of Auckland, New Zealand

## Abstract

The motion of ions, molecules or proteins in dendrites is restricted by cytoplasmic obstacles such as organelles, microtubules and actin network. To account for molecular crowding, we study the effect of diffusion barriers on local calcium spread in a dendrite. We first present a model based on a dimension reduction approach to approximate a three dimensional diffusion in a cylindrical dendrite by a one-dimensional effective diffusion process. By comparing uncaging experiments of an inert dye in a spiny dendrite and in a thin glass tube, we quantify the change in diffusion constants due to molecular crowding as *D_cyto_/D_water_* = 1/20. We validate our approach by reconstructing the uncaging experiments using Brownian simulations in a realistic 3D model dendrite. Finally, we construct a reduced reaction-diffusion equation to model calcium spread in a dendrite under the presence of additional buffers, pumps and synaptic input. We find that for moderate crowding, calcium dynamics is mainly regulated by the buffer concentration, but not by the cytoplasmic crowding, dendritic spines or synaptic inputs. Following high frequency stimulations, we predict that calcium spread in dendrites is limited to small microdomains of the order of a few microns (<5 μm).

## Author Summary

Diffusion is one of the main transport phenomena involved in signaling mechanisms of ions and molecules in living cells, such as neurons. As the cell cytoplasmic medium is highly heterogeneous and filled with many organelles, the motion of a diffusing particle is affected by many interactions with its environment. Interestingly, the functional consequences of these interactions cannot be directly quantified. Thus, in parallel with experimental methods, we have developed a computational approach to decipher the role of crowding from binding. We first study here the diffusion of a fluorescent marker in dendrites by a one-dimensional effective diffusion equation and obtained an effective diffusion constant that accounts for the presence heterogeneity in the medium. Furthermore, comparing our experimental data with simulations of diffusion in a crowded environment, we estimate the intracellular calcium spread in dendrites after injection of calcium transients. We confirm that calcium spread is mainly regulated by fixed buffer molecules, that bind temporarily to calcium, and less by the heterogeneous structure of the surrounding medium. Finally, we find that after synaptic inputs, calcium remains restricted to a domain of 2.5 *µ*m to each side of the input location independent of the input frequency.

## Introduction

Dendrites of neurons contain a complex intracellular organization made of organelles, such as mitochondria, endoplasmic reticulum, ribosomes and cytoskeletal network generated by actin and microtubules [1]–[3]. The cell cytoplasm is thus a crowded rather than diluted medium in which diffusional mobility of small molecules is restricted [3]–[7]. Molecular crowding can affect many biochemical processes such as, protein folding [8]–[10], enzymatic reactions [11]–[13] and signal transduction [14]. Although electromicroscopy images [15] reveal the complexity of dendritic organization, there are no direct methods to estimate the functional consequence on diffusion. Modeling in combination with Monte-Carlo methods [16]–[19] allowed to study diffusion in crowded media. Depending on the size of the diffusing molecule and the interactions with the heterogeneous media, crowding can lead to anomalous or normal diffusion [4], [20]–[24].

Neuronal calcium is an fundamental and ubiquitous messenger [25], [26]. It is regulated by cytoplasmic crowding, mobile and immobile calcium buffers [27]–[30], pumps and dendritic spines, which cannot be easily dissociated experimentally. It was already noticed and quantified [31] that cellular calcium buffers can determine amplitude and diffusional spread of neuronal calcium signaling. Precisely, fixed calcium buffers tend to retard the signal and to lower the apparent diffusion coefficient, whereas mobile buffers can contribute to calcium redistribution. To study calcium dynamics, we develop in the first part, a model of diffusion in a crowded three-dimensional dendrite, that we reduce to a one-dimensional effective diffusion process. The model is general and can be applied to protein diffusion in membranes or in endoplasmic reticulum-like networks [16], [32]. In a second part, we use uncaging experimental data of an inert dye (fluorescein) in a spiny dendrite and in a glass tube of similar size filled with aqueous solution to estimate the reduction of the diffusion constant in a dendrite. These experiments are repeated by Brownian simulations in a 3D model dendrite in order to validate our one-dimensional model.

In the last part, we use the previously derived effective diffusion constant and simulate a system of reaction-diffusion equations in one dimension to study calcium dynamics in a dendrite. We accounted for calcium buffers, pumps, dendritic spines and synaptic inputs. We show that for moderate organelle crowding, calcium spread is mainly restricted by the buffer and the pump concentration and not by obstacles or dendritic spines. Although crowding restricts dendritic diffusion by a factor 20, it is not responsible for the high calcium compartmentalization (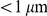) in dendrites [33], [34]. We further show that following high frequency stimulations, calcium spread does not exceed 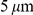. In summary, calcium microdomains are highly regulated by various active processes such as calcium buffers, pumps and stores.

## Results

Our results are divided into three sections. In the first section, we present the diffusion model for an inert dye in a crowded dendritic medium. The model is derived from a periodic compartmentalization of the dendritic domain. It is followed by an extension of the model to almost periodic compartments and the analysis of the mean time a particle takes to travel across the dendrite. In the second part, we present the outcome of the uncaging experiments of fluorescein to probe the dendritic medium and to estimate the model parameters. It is followed by a comparison to Brownian simulations, which repeat these experiments on a computer. Finally, we provide mean-field simulation results for calcium spread in a dendrite under the additional presence of stationary buffers, pumps and synaptic input.

### Crowding model

#### Modeling diffusion in a heterogeneous dendritic cytoplasm

**Figure 1:**
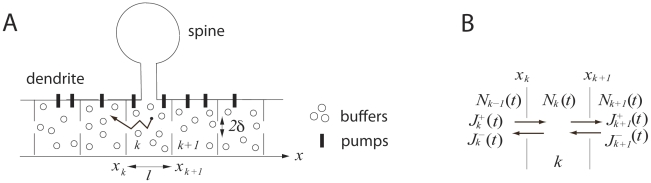
Compartmentalized model dendrite with attached spine including buffers and pumps. The model dendrite is organized as a sequence of periodic compartments of length 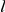. The compartments are connected through little openings of radius 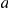 where molecules can pass to neighboring compartments. (B) Inward and outward fluxes through the small openings of compartment 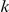 used in the derivation of the effective diffusion equation.

To characterize diffusion in a heterogeneous dendrite, containing various organelles such as mitochondria, spine apparatus, endoplasmic reticulum and other structures, we propose to derive from a three dimensional analysis a one-dimensional effective diffusion equation. In the limit where the space in between organelles is small, particles can still move inside a dendritic domain 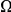 and the nature of the motion is not impaired, and is well approximated by the Smoluchowski limit of the Langevin equation [35]: a particle at position 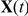 at time 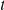 is described by
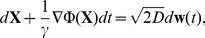
(1)where 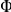 is a potential per unit of mass, 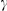 is the friction coefficient, 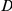 is the aqueous diffusion constant and 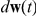 is Gaussian white noise. The potential 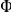 represents the effective force on the particle. When a moving molecule hits impenetrable organelles 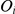, it is reflected. The distribution of independent molecules is characterized by the probability density function (pdf) 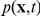 which satisfies the Fokker-Planck equation
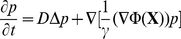
(2)in the domain 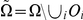, and a zero flux condition on the organelles and the dendritic membrane 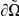:
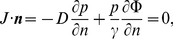
(3)where 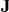 is the flux and 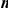 the outer normal of the domain 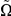. To study the overall effect of crowding on the diffusion, we shall approximate equations (2) and (3) by deriving a one-dimensional effective diffusion equation along the dendrite. We adopt an approach based on a compartmentalization of the dendritic domain and the small hole theory [36], which provides the mean time for a Brownian particle to exit a domain through a small absorbing opening. This method allows us to obtain an explicit expression for the apparent diffusion constant. We divided a dendrite into periodic compartments of length 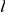 and volume 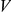, (Figure 1A) separated from their neighbors by a reflecting cross section, except for a small opening of radius 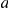. This compartment should be large enough so that the organelle density is the same in each of them. The small openings allow diffusing molecules to move across compartments. In contrast to previous models where crowding has been described by spherical obstacles [37] that pose barriers to diffusing molecules, we model crowding as the sequence of periodic compartments and small openings at the boundaries of neighboring compartments. A compartment 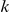 starts at position 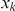 and ends at position 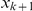 (Figure 1B). The number 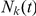 of particles in compartment 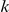, changes according to the net flux across the small windows. The flux can be estimated by the small hole approximation for the Mean First Passage Time (MFPT) 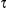 a Brownian particle takes to escape a small opening [36], [38]–[41]). At first order in 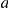, 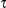 is approximated by
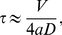
(4)where 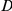 is the aqueous diffusion constant and 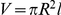 the cylindrical compartment volume. 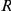 denotes the dendrite radius. Note that the MFPT solely depends on the ratio 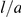 for fixed radius 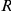. From numerical studies (data not shown) we find that formula (4) holds for 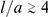 to reasonable accuracy (relative error 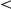
0.05). In the long-time asymptotic regime (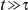), the unidirectional flux of particles through a small hole is 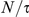. The net flux is the difference between the unidirectional fluxes in opposite direction, and thus, given by
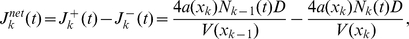
where we have assumed that the size of the opening and the compartment volume may be spatially dependent. The conservation of mass imposes that the changes in the number of particles inside the compartment 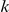 is the sum of the net fluxes at position 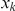 and 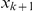 (Figure 1B) and thus
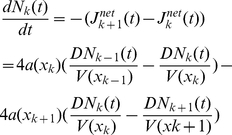
(5)Using a Taylor expansion with 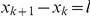 for a fixed value of the length 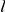, equation (5) becomes
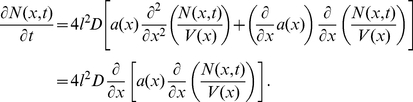
(6)


Introducing the concentration 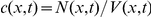 we obtain
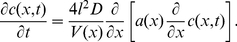
(7)Similar equations have been derived in other contexts [42]–[44]. If the parameters 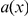 and 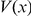 are spatially independent, equation (7) simplifies to
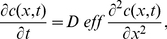
(8)where the effective diffusion constant 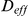 is given by
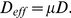
(9)


The compartment parameter is
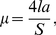
(10)where 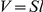 and 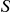 is the cross-sectional area. The effective diffusion constant depends on two parameters: the compartment length 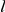 and the size of the opening 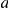. We determine the model parameters by (i) measuring the ratio of diffusion constants 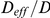 and (ii) a calibration condition of the form 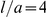. The latter condition is chosen such that the small hole approximation (4) is valid to reasonable accuracy (relative error 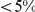), which we have tested in numerical simulations (not shown here). The effective diffusion constant for spatially homogeneous compartments is given by 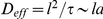. Thus, the calibration condition sets one parameter arbitrarily within the limits of the small hole approximation and measurements of the diffusion constant will fix the other parameter. Equation (6) can be associated with a stochastic equation
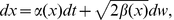
(11)where the drift and diffusion terms are
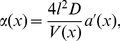
(12)

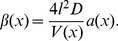
(13)(a prime denotes differentiation with respect to 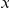). Thus, the drift disappears for spatially homogeneous opening sizes between compartments (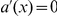).

The previous analysis can be applied to the motion of receptors on the surface of neurons, which contains impenetrable micro domains [45]. When the surface can be decomposed into a set of compartments containing small openings, we can apply the results of the small hole computation derived in dimension two [36], [38]: the mean time for a Brownian molecule to escape a domain of area 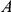 through a small hole is approximated by
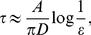
(14)where 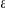 is the ratio of the absorbing to the total length of the two dimensional compartment. Following the same reasoning as in the previous paragraph, the receptor density satisfies the one dimensional reduced equation
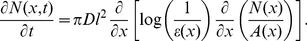
(15)


#### Crowding model for almost periodic diffusion barriers

To further analyze the effect of diffusion barriers, we investigate how our previous analysis is affected by an almost periodic distribution of barriers, where a random jitter is modelled as white noise. We will see that diffusion in such medium is characterized by a fourth order diffusion equation. This analysis shows that approximating diffusion by dimensional reduction can lead to a none-classical diffusion description. We start with a compartment position 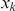 given by
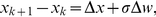
(16)where 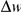 is a centered Brownian variable of variance 1 and the drift is a fixed number 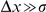. When the other parameters 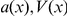 are spatially independent, the conservation of mass leads for compartment 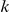 to
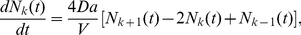
(17)where 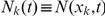. It can be shown that by expanding the functions 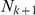 and 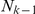 in terms of the random position 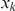 the mean number of diffusing molecules is given by a fourth order diffusion type equation
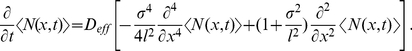
(18)


The effective diffusion equation (8) is recovered in the limit 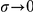. In the small jitter limit 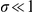, the effective diffusion constant reduces to
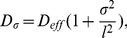
(19)where 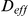 is defined in (9). We conclude that jittering leads to an increase in the diffusion constant compared to a periodic arrangement of barriers. Interestingly, the distribution of compartments affects the nature of the apparent diffusion process: in the periodic case, the apparent diffusion is described by the standard second order diffusion while fluctuations in the compartment distribution lead to an apparent diffusion that is described by a fourth order equation.

#### Mean time for a diffusing particle to travel across a dendrite

A possible application of the previous theory and equation (6) is to estimate the mean time 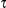 for a diffusing particle, such as a transcription factor, to travel across a nonbranching dendrite.

The probability density function to find a molecule at position 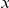 at time 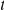 is 
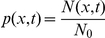
, where 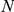 is the number of molecules per unit length. We can apply the standard theory of first passage time [35] to equation (6) and obtain an equation for the mean first passage time 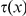:
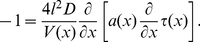
(20)


To obtain the MFPT, 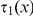, to reach the cell body (soma) from any starting point, we solve equation (20) in a dendrite sealed at the distance 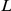 (Reflecting boundary condition) from the nucleus,
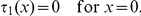


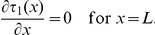



The solution is
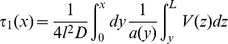
(21)For example, when 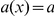, and the compartment volume is constant 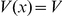, the mean time a diffusing molecule takes to travel from location 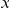 to the nucleus is given by
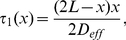
(22)where the effective diffusion constant is defined in (9). Similarly, the MFPT, 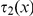, in opposite movement direction, i.e., from the (reflecting) soma to an absorbing site at 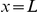 in the dendrite, is given by
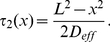
(23)


We conclude that in a dendrite with an effective diffusion constant of 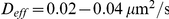 [46], and 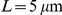, the mean time for a mRNA to reach the soma, starting from the tip (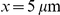) is about 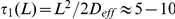 min.

### The effect of crowding in dendrites

#### Uncaging experiments in a dendrite

**Figure 2:**
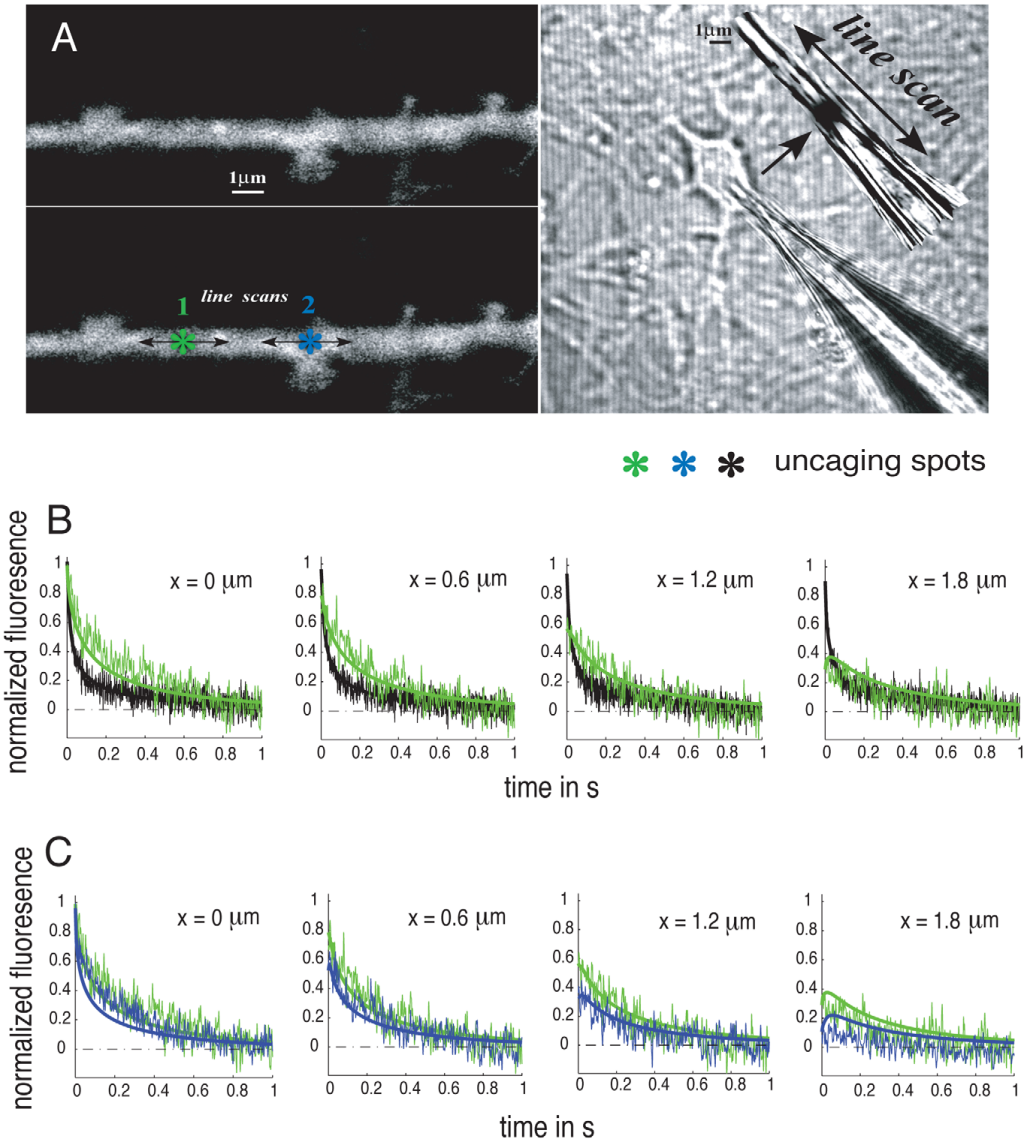
(A) Images of the dendritic segments and the glass pipette used in the experiments. The sites of the uncaging spots are indicated. (B) Fluorescein transients in the pipette (black) and in the dendritic medium far away from any attached spine (green) at different distances from the uncaging spot. (C) Fluorescein transients in the dendrite near and far away of any attached dendritic spine are shown in blue and green, respectively. Fluorescein was uncaged at the base of the spine at location 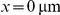. The data are averaged values over several uncaging experiments (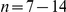). The numerical solutions of the 1D effective diffusion equation are shown as solid lines.

To study crowding inside a dendrite, we use a set of experiments in which we measure the diffusion time course of a caged inert dye molecule fluorescein (Materials and Methods). To estimate the effect of crowding in the dendritic medium, we compare the diffusion time course near and far away from any dendritic spines (to avoid any perturbation by the spine domain) to diffusion in a glass pipette of a similar radius. Figure 2A shows confocal microscopy images of a dendritic segment with several attached spines and the glass pipette. We first compare the fluorescent transient in the dendrite and in the glass tube at different locations from the uncaging spot (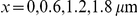). We find a much faster decay in the aqueous solution of the pipette compared to the dendrite (Figure 2B). The fluorescent signals were averaged over several uncaging experiments (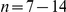). The diffusion constants were extracted by a least-square fit of the data to the numerical solutions of equation (8), which consider a spine as homogeneous at the length scale of the compartment length 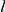.

For the pipette data, where fluorescein diffuses freely, this method led to a diffusion constant of 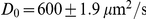, whereas in the dendritic medium far away from any attached spines, we estimated a diffusion constant of 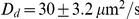. This number is not far from the upper estimate obtained for axoplasm of metacerebral cells of Aplysia california, where 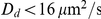 [31]. We conclude that cytoplasmic crowding in the dendrite resulted in a drastic reduction of the apparent diffusion constant by a factor of 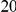. Using the crowding model presented in the previous section, we can estimate the compartment length 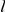 and the opening size 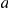 that leads to this reduction in the diffusion constant. From formula (9) and the calibration condition, we find that 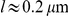 and 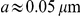, where the dendritic radius was set to 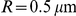.

We further investigated the influence of spine on dendritic diffusion: we initiated a dye transient in the dendritic shaft at the base of a spine by uncaging fluorescein. Figure 2C shows the fluorescent signals in the presence and absence of the spine at different locations from the uncaging spot. We obtain a slightly larger diffusion constant near a dendritic spine 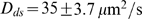 compared to no spine.

#### Brownian simulations of the uncaging experiments

**Figure 3:**
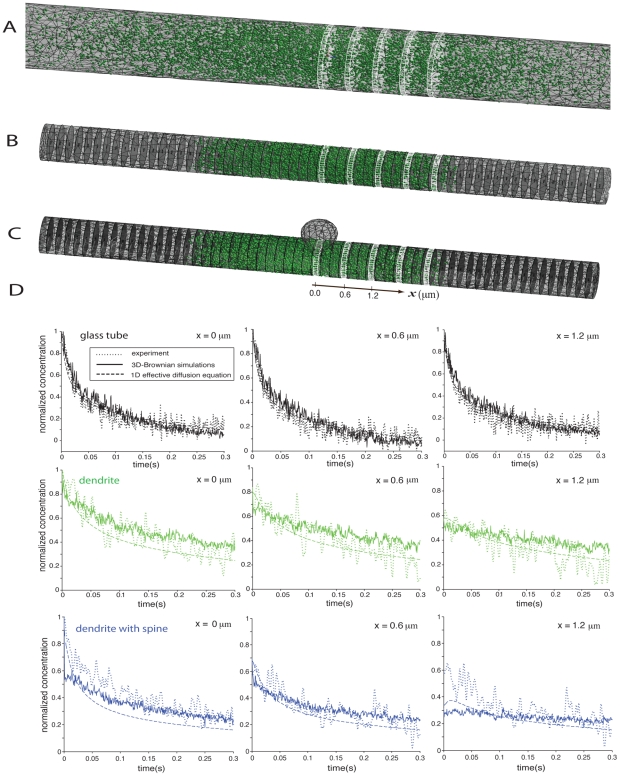
Brownian simulations of uncaging experiments. (A) Model glass pipette (radius 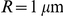 and length 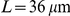). Shown is the initial particle distribution as taken from the experimental data and the sampling volumes (white cylindrical disks) at different locations from the uncaging spot (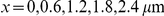). (B) Compartmentalized model dendrite (radius 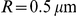 and length 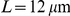). The compartment length and the opening size are derived from the theoretical model (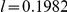 and 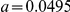). (C) Compartmentalized model dendrite with attached spine (dendrite geometry as in B with spine neck radius: 0.3 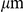, spine neck length 0.2 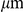, spine head radius 0.4 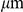). (D) Comparison of 3D Brownian simulations with the uncaging experiments and the results derived from the solutions of the 1D effective diffusion equation. The normalized concentration profiles are shown for the glass tube (A), the dendrite (B) and the dendrite with attached spine (C) at three locations from the uncaging spot (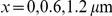).

To support our modeling approach we use Brownian simulations (Figure 3) to reproduce the uncaging experiments in a glass pipette (Figure 3A) and in a 3D cylindrical model dendrite far (Figure 3B) and near a dendritic spine (Figure 3C). The methods used for the implementation of the Brownian simulations are described in Materials and Methods.

We first calibrate the parameter of the model: according to equation (9), a reduction of diffusion constants by a factor of 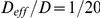 results in a compartment length of 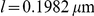 and an opening size of 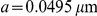. The spine characteristic lengths are taken from the confocal microscopy image Figure 2A. We simulated 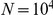 particles (of fluorescein) and sampled the concentrations in cylindrical disks (height = 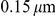) at locations of the experimental recording sites (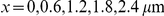) for a duration of 0.7 ms, which corresponds to the temporal resolution of the experimental data. For the simulations in the glass pipette and in the dendrite near and far any attached spine, the initial distribution in the axial direction was taken from the experimental data, whereas in the radial direction, it is taken to be homogeneous. The diffusion constant of fluorescein in aqueous solution was set to 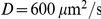 in all simulations. Figure 3D shows the comparison of the 3D Brownian simulations with the experifmental data and the results derived from the 1D effective equation. Note that in all simulations the concentration at 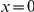 and 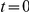 is normalized to 1. There is a sharp drop of particle concentration in the case of a dendrite with attached spine at 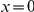. This is due to the flux of particles out of the sampling box into the spine. Note further that the 1D effective diffusion is only valid in the long-time asymptotic regime where 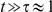 ms. We conclude that the results of the 1D effective diffusion equation and the 3D Brownian simulations in our diffusion model recover the time course of the experimental data, confirming our overall our approach. A movie (Video S1) of our Brownian simulations in the dendrite with an attached spine is given in the Text S1.

### Calcium dynamics in crowded dendrites

In addition to cytoplasmic crowding, calcium dynamics is regulated by many factors such as binding to buffer molecules (e.g., calmodulin and calcineurin), dendritic spines and various types of pumps located on the dendritic surface (PMCA, NCX) and on the surface of internal organelles such as the endoplasmic reticulum (SERCA). It is usually not possible to dissect experimentally the contribution of each process, and we shall apply our previous result to study calcium spread in dendrites.

We present a reaction-diffusion equation (Materials and Methods) to simulate calcium dynamics in both spiny and aspiny dendrites. At this stage, we do not take into account the intracellular calcium stores, and thus, we exclude the generation of calcium waves through CICR, nor do we model spontaneous dendritic calcium spikes or calcium transients associated with back-propagating action-potentials. We shall focus here on the local spread of calcium transients and we ignore global calcium events. We include in our simulations the effect of buffers, pumps, spines and synaptic input. The contribution to calcium dynamics for each active component is provided in the Materials and Methods.

### Calcium is highly restricted by the buffer activity and not by molecular crowding

**Figure 4:**
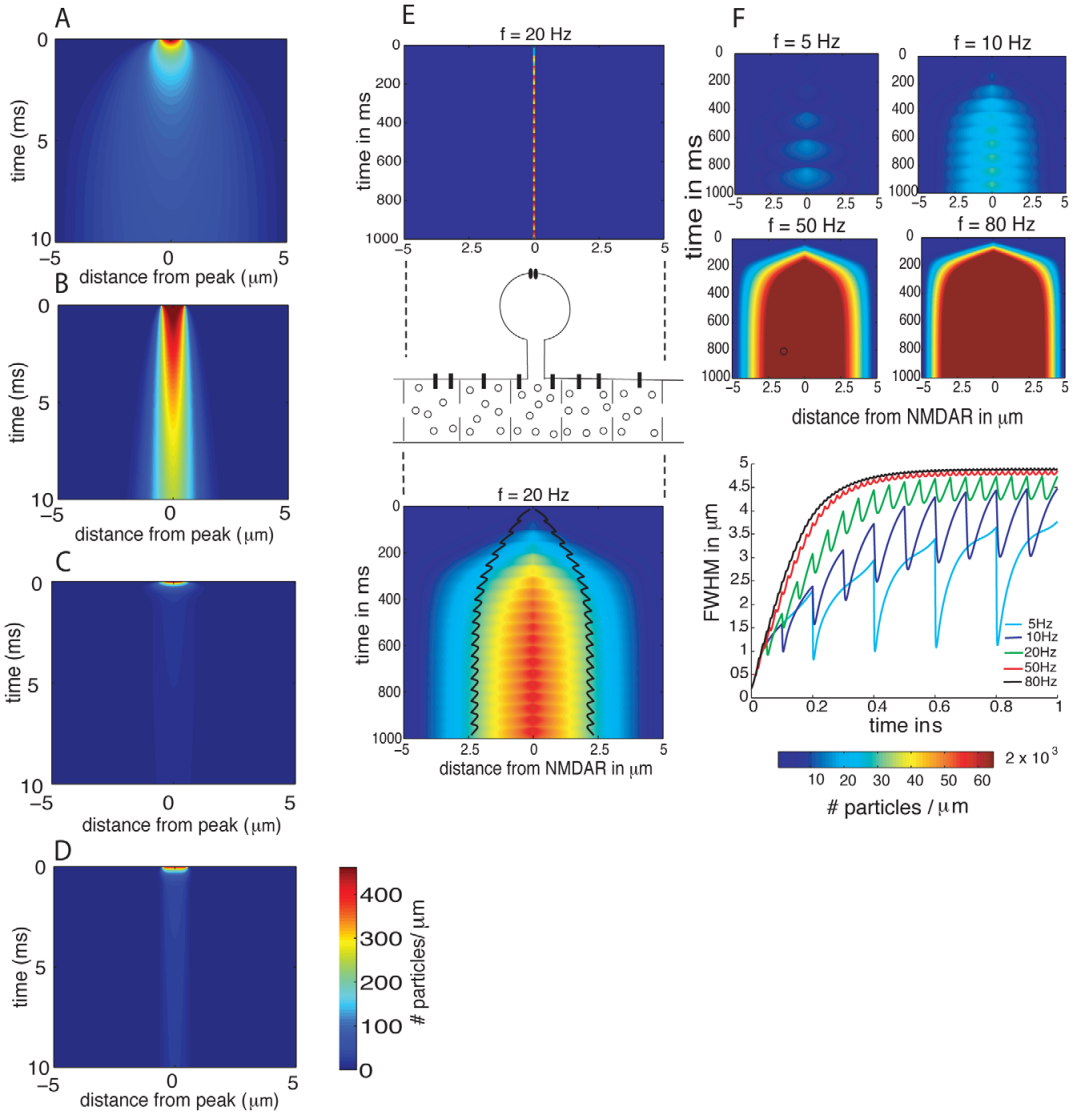
(A) Calcium diffusion in an aqueous solution contained in a pipette of length 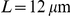. (B) Calcium diffusion in a crowded dendrite with an effective diffusion constant of 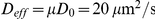. A calcium transient of 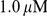 was initiated at 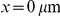. Note that the initial concentration is equal to about 600 particles per 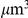 and evaluates to about 470 particles per micron for a dendrite with diameter 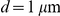. (C) Same settings than in (A) but with additional buffers (medium buffer concentration) and pumps. (D) Same settings than in (B) but with additional buffers (medium buffer concentration) and pumps. (E) 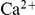-influx was injected at 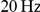 for 1 s at the location of the NMDAR in the middle of the dendritic segment as shown in the upper and middle panel. The resulting spatiotemporal 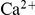-profile in the dendrite is shown in the lower panel. (F) Spatiotemporal profiles of 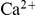 in the dendrite for different influx frequencies at the location of the NMDAR. (G) Corresponding calcium spread in the dendrite as measured by the full width at half maximum (FWHM) of the calcium signal.

We first simulated calcium diffusion in an aqueous solution (contained in a glass pipette) by initiating a calcium transient and solving the one dimensional diffusion equation (41)–(45) with a diffusion constant of 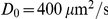 (Figure 4A). The effect of crowding alone on calcium diffusion in a dendrite was simulated by reducing the free diffusion constant to 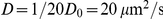 (Figure 4B). We assume here that the effects of crowding on motion are the same for fluorescein molecules and calcium ions attached to a dye molecules. As expected, crowding leads to a more localized and persistent calcium transient compared to free diffusion in an aqueous solution.

We next added two types of imobile buffers, calmodulin (CaM) and calcineurine (CN), as well as pumps (NCX and PCMA) to the simulation. The buffer concentration was varied between low ([CaM]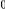: 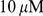, [CN]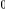: 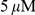), medium ([CaM]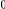: 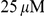, [CN]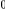: 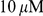) and high ([CaM]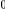: 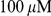, [CN]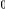: 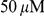) levels. Figure 4C and D show the effect of fast buffering on calcium dynamics in aqueous solution and in a crowded dendrite, respectively, for medium buffer concentration. The differences are small. The calcium signal in the crowded medium is more localized in space and slightly longer lasting than in aqueous solution. From these simulation results, we conclude that the spatiotemporal extent of the calcium signal is highly restricted by the stationary buffer activity. These results agree qualitatively with other uncaging experiments of calcium in glass tubes and dendrites [47].

### Calcium spread following a large range of frequency stimulation is less than 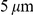 around the source

We next analyze calcium spread originating from localized inputs such as synapses. At dendritic synapses calcium can enter through NMDA-receptors. To estimate calcium spread as a function of the synaptic input frequency, we simulated 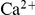-influx in the middle of a dendritic segment (Figure 4E). Buffers and pumps were set to their default values (Table 1). We initiated calcium transients in the crowded model dendrite for different input frequencies (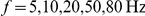). The spatiotemporal extent of the calcium signal for different input frequencies is given in the intensity plots Figure 4F. Calcium spread is measured by the full width at half maximum (FWHM) of the calcium signal. Interestingly, for input frequencies larger than 20 Hz, the calcium signal in the dendrite reaches a stationary value. For high input frequencies (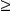
20 Hz) calcium spread does not exceed 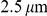 ( = 0.5
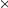
FWHM) as measured from the input source. This is in agreement with the experimental data where calcium spread was contained within a domain of about 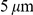. We conclude that buffer and pumps limit calcium spread to few micrometers.

**Table 1:** Model parameters.

Parameter		Value	Reference
Glass tube geometry			
	length of glass tube 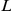	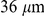	adjusted
	glass tube diameter 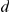	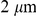	adjusted
Dendrite geometry			
	length of dendritic segment 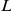	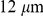	adjusted
	dendrite diameter 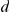	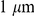	(Koch, 1999)
	dendritic cross section 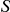	0.785 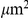	adjusted
Crowding			
	compartment length 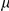	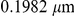	adjusted
	opening size 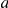	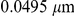	adjusted
	compartment parameter 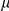	0.05	adjusted
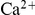 -ions			
	diffusion constant of free 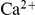 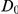	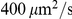	(Korkotian et al., 2004)
	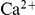 initial concentration for pulse 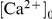	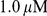	(Korkotian et al., 2004)
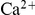 -pumps			
	pump rate for PMCA 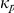	0.27 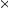 10 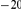 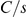	(Erler et al., 2004)
	pump density for PMCA 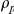	9200/ 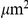	(Erler et al., 2004)
	half-saturation constant for PMCA 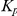	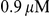	(Korkotian et al., 2004)
	hill coefficient for PMCA 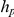	1.0	(Stauffer et al., 1995)
	pump rate for NCX 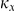	0.48 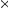 10 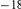 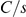	(Erler et al., 2004)
	pump density for NCX 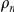	300/ 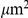	(Erler et al., 2004)
	half-saturation constant for NCX 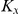	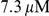	(Fujioka et al., 2000)
	hill coefficient for NCX 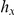	1.7	(Fujioka et al., 2000)
Calmodulin			
	total concentration 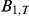	10, 25 (default), 100 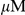	(Volfovsky et al., 1999)
	forward binding rate for 1st binding 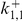	160 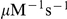	(Johnson et al., 1996)
	backward binding rate for 1st binding 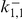	405 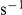	(Johnson et al., 1996)
	forward binding rate for 2st binding 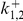	160 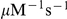	(Johnson et al., 1996)
	backward binding rate for 2st binding 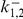	405 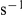	(Johnson et al., 1996)
	forward binding rate for 3st binding 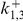	2.3 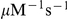	(Johnson et al., 1996)
	backward binding rate for 3st binding 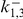	2.4 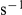	(Johnson et al., 1996)
	forward binding rate for 4st binding 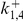	2.3 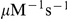	(Johnson et al., 1996)
	backward binding rate for 4st binding 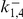	2.4 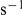	(Johnson et al., 1996)
Calcineurine			
	total concentration 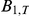	5, 10 (default), 25 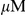	(Volfovsky et al., 1999)
	forward binding rate 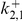	50 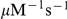	(Volfovsky et al., 1999)
	backward binding rate 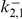	25 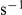	(Volfovsky et al., 1999)
Calcium dye (Fluo-4)			
	total concentration 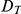	2 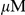	(Korkotion et al., 2004)
	forward binding rate 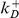	60 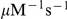	(Korkotion et al., 2004)
	backward binding rate 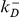	170 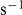	(Korkotion et al., 2004)
NMDA-R			
	current through a single NMDAR 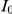	9 pA	(Pina-Crespo and Gibb, 2002)
	fraction of current carried by 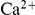 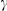	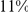	(Burnashev, 1995)
	time constant (decay) 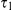	80 ms	(Zador and Koch, 1994)
	time constant (rise) 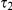	3 ms	(Zador and Koch, 1994)
	radius of receptor 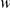	0.025 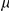 m	adjusted
Spines			
	spine radius 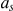	0.05–0.16 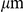	(Koch, 1999)

Parameters used in the stochastic simulation experiments and mean-field calcium dynamics simulations.

## Discussion

### Calcium spread in crowded dendrites

We have shown here that dendritic crowding reduces the diffusion constant of inert Brownian molecules by a factor of 20 when compared to diffusion in an aqueous solution. We have used this result to estimate calcium spread in dendrites. We found that in the absence of regenerative mechanisms (VSCC, calcium stores), the spread of calcium largely depends on the buffer concentration and moderate molecular crowding does not play a significant role in shaping calcium dynamics. Thus, crowding has only a minor effect compared to the cumulative effect of pumps and buffers. In addition, the presence of a single (passive) spine at the location of calcium release did not influence calcium diffusion in the dendrite.

In this study, we have analyzed the effect of molecular crowding on calcium spread under the presence of stationary buffers. Assuming that the diffusion constant of calcium and fluorescein are reduced by the same factor due to the effect of molecular crowding, our results confirm previous studies that calcium spread is largely restricted by the effect of stationary buffers [31], [48]–[50]. Our analysis showed only a small effect of molecular crowding on calcium spread (Figure 4C and 4D): slightly more calcium molecules were bound to buffers in the crowded condition.

These results are qualitatively consistent with stochastic simulations in a cubic cell model under different crowding and buffer mobility conditions [19], where it has been shown that molecular crowding affects the calcium signaling system mainly through crowding-induced binding of calcium to buffer molecules and less through the direct hindrance of calcium diffusion. This study showed further that these effects are not additive. Interestingly, the reduction in diffusion constant due to molecular crowding was found to be 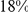 for moderately crowded environments with 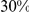 excluded volume fraction. In our study, the reduction of the calcium diffusion constant was extrapolated from fluorescein uncaging experiments in the dendritic medium, which resulted in a much higher value. This difference might result from additional crowding effects such as cavities that were not modelled in the stochastic simulations.

### Calcium is restricted in microdomains near each synaptic input

Calcium microdomains have been observed during spontaneous and electrically evoked activation of synapses on dendritic shafts in aspiny neurons [34]. Compartmentalization into domains of about 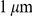 resulted from fast kinetics of calcium permeable AMPA receptors and fast local extrusion via the 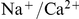 exchanger [34]. In general, as observed in Figure 4, calcium spread is robustly confined in a domain of less than 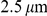 from the input source and this seems to be independent of the synaptic firing frequency. Thus, calcium dynamics seems to be well regulated by buffers, stores and extrusion mechanisms.

It is certainly a requirement for dendrites to prevent calcium spread over large distances because it is not only the primary messenger in the induction of synaptic plasticity, such as long term potentiation (LTP) [51], but it is also involved in morphological changes and in the regulation of receptor trafficking such as AMPA [52]. While organelle localization might depend on the dendritic local needs (protein syntheses, energy supply and local calcium stores), calcium pump densities and calcium buffer concentrations might be regulated independently to maintain calcium homeostasis. It remains an unsolved question to determine how pumps and calcium buffer molecules are regulated along a dendrite.

### Molecular trafficking near dendritic spines

Using our previous computations, we found that (passive) dendritic spines in this mean-field approach do not contribute much in dendritic calcium regulation (data not shown). In general, our result suggests that spines should not significantly affect the movement of diffusing particles along the dendrite. However, in the case of calcium, we have not taken into account a possible calcium propagation through the endoplasmic reticulum network, which may lead to a very different type of propagation.

Dendritic spines can be seen as the ultimate place of confinement in dendrites: indeed, calcium exchangers located on the endoplasmic reticulum surface or on the spine neck membrane can prevent calcium from diffusing into the spine head [53], [54]. In addition, large crowding observed at the spine base due to various types of organelles such as the endoplasmic reticulum or the spine apparatus [2], [15] can prevent diffusing molecules from entering the spine neck. However, it is not clear whether mRNA or transcription factors can enter dendritic spines by passive diffusion or whether active processes are required.

## Materials and Methods

### Fluorescein experiments in dendrites

Cultures were prepared as detailed in [47]: we use wistar rat pups at P1. Hippocampal tissue was mechanically dissociated and plated on 12 mm glass coverslips at 3–4
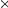
105 cells per well in a 24 well plate. Cells were left to grow in the incubator at 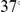
C, 5% CO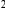 for 4 days, at which time the medium was changed to 10% HS in enriched MEM. The medium was changed four days later to 10% HS in enriched MEM. Cells were transfected at 1 wk in culture with DsRed plasmid to visualize the dendrites and spines using a lipofectamine 2000 (Invitrogen) method. On the day of imaging, the glass was transferred to the recording medium containing (in mM): NaCl 129, KCl 4, MgCl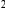 1, CaCl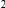 2, glucose 10, HEPES 10, and TTX 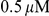. pH was adjusted to 7.4 with NaOH, and osmolarity to 315 mOsm with sucrose. Ten-fourteen day old cultured cells were patch clamped at the soma and recorded with a glass pipette containing (in mM): K-gluconate 140, NaCl 2, HEPES 10, EGTA 0.2, Na-GTP 0.3, Mg-ATP 2, phosphocreatine 10, and 100 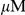 of caged fluorescein (Molecular Probes) at pH 7.4 having a resistance of 6–12 M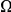. Signals were amplified with Axopatch 200 (Axon Instruments Inc. Foster City, CA). Cells were imaged with a 63× water immersion objective (NA = 0.9). UV laser was aimed at a spot of 1 m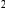 in the center of the field of view. A line scan mode (0.7 msec/line) was used along an imaged dendrite to measure fast changes in fluorescence following flash photolysis of caged fluorescein. In the second stage of the experiment, the content of patch pipettes, containing caged fluorescein, was sucked out and introduced into additionally prepared pipettes with long and sharp tips, having tens of microns in length and about 1–2 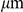 in diameter making their geometry similar to a “typical” dendrite. Same line scan mode was used to compare changes in fluorescence in a dendrite and in a glass tube, containing similar concentrations of caged fluorescein. Data were analyzed using custom made MATLAB-based programs. Steps of 0.6 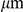 from the center of the uncaging sphere were defined through the line scans and pixels inside every step were horizontally averaged. Every line scan trial was repeated 7–14 times. Statistical comparisons were made with t-tests.

### Brownian simulations

We implemented the Brownian simulations in MATLAB using a ray-tracing algorithm. To overcome the huge computational burden that Brownian simulations in complex domains impose, we made heavily use of MATLAB's object-oriented programming and vectorization features as well as of the external C/C++ interface functions capabilities (MEX-files). We first constructed a triangular mesh of the simulation domain (e.g., cylinder, cylinder with spine, see Figures 3 A,B) using a simple mesh generator based on distance function (DistMesh package, [55]). The (meshed) simulation domain was then equipped with user-defined sampling boxes, an initial distribution of particles and diffusion barriers (e.g., disks with small holes, see Figures 3 B,C). We predefined a sampling interval (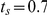 ms) at which the particle concentrations in the sampling boxes were measured.

Surface mesh elements were defined to be either reflective or absorbing. The top and the bottom of the cylindrical domain was set to be absorbing while all other surface elements were defined to be reflective. Particle rays crossing reflecting boundaries or obstacles were reflected according to the law of light reflection. To speed up the code we divided the simulation domain into partition voxels. For each partition voxel a list of contained objects (mesh elements, obstacles) was pre-computed and provided to the algorithm during execution.

The Brownian simulation was implemented using an Euler-scheme with adaptive-step size. Steps were defined by the distance to mesh elements and obstacles. The closer the particles were to objects the smaller the step size was chosen. As a rule of thumb, the minimal step size was determined by 0.3–0.5 of the smallest length scale that had to be resolved (e.g., the radius of the hole of the disks, see Figure 3 B). The (vectorized) particle rays were traced in the voxels and tested for intersections with mesh elements or objects. If intersections occurred the particles were either reflected or absorbed. It is important to note that an adaptive-step size algorithm leads for each particle to a different progress in physical time. Hence, the measurement of particle concentrations at fixed sampling times, required the implementation of a scheduler that removed particles temporarily from the simulation and stored their positions. Our simulations lasted between several hours to several days on a cluster depending on the number of particles, number of objects and the minimal step size. We have made extensive use of MATLAB's visualization tools to monitor the simulations and to generate visual outputs of the simulation results (see snapshots in Figures 3 A–C and a movie (Video S1) in the Text S1). We have included in the Text S1 a validation study of diffusion in a cylindrical domain with absorbing boundaries at the top and bottom. Different measures such as global and local particle concentrations as well as the mean first passage time to the absorbing boundaries are extracted from the simulations and compared with existing analytical results. The test-simulation is shown in Video S2. A good agreement between these results was obtained, and thus, evidence for the correctness of the implemented algorithm in the Monte-Carlo simulation tool is provided.

### Calcium dynamics

The spatiotemporal calcium signal in the dendrite is regulated by several active and passive components that are described next.

#### Calcium buffers

Dendrites contain a large number of different buffers. The reactions of a buffer 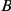 that can bind 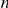 calcium ions is modeled by the series of chemical reactions
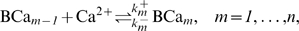
(24)where 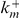 and 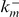 are the forward and backward rates for 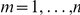, respectively. We choose two representative members of the buffer molecules: calcineurin (CN) with one calcium binding site (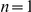) and calmodulin (CaM) with four binding sites (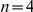). The kinetic equations are derived from the standard theory of chemical reactions, leading to a coupled set of odes for the unknown calcium concentrations, [Ca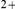] and buffer concentrations, [BCa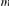], with 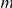, (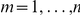), calcium bonds:
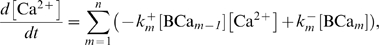
(25)

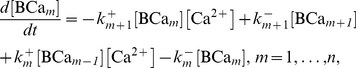
(26)

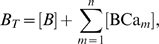
(27)In the following we will not use concentrations as the dynamic variables, but the number of particles (in 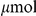) per unit length, 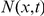. The conversion from calcium concentration to particles per unit length is
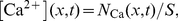
(28)where 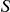 is the cross section of the dendrite.

#### Calcium pumps

Two basic mechanisms are responsible for the removal of calcium ions across the neuron membrane: the ATP-driven plasma membrane 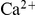 pumps (PMCA) and the 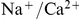 exchanger (NCX). The PMCA pumps extrude 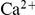 ions against the concentration gradient using the energy provided by the ATP molecules. The sodium-calcium exchanger can move one calcium ion inwards for moving three sodium ions outward. Both extrusion mechanisms are described by similar equation: the loss of calcium ions through the PMCA pumps (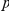) and NCX (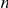) is modeled according to
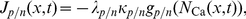
(29)with an activation characteristics
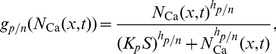
(30)where the half-saturation concentration is 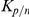, the extrusion rate per pump (number of ions per unit time) is given by 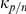, the density of pumps per unit length is denoted by 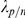 and 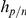 is the hill coefficient.

#### Passive effect of dendritic spines

Dendritic spines are modeled as passive calcium absorbers. In our model, calcium ions entering a dendritic spine are totally absorbed. The flux of calcium ions into the dendritic spine depends on the spine neck radius. It can be computed in the configuration where the dendrite is compartmentalized and the compartments are connected through small openings (Figure 1A). In that case, the openings between compartment and the spine entrance are well separated, and thus the flux of calcium ions into a dendritic spine with spine neck radius 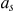 located at a longitudinal position 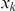 is
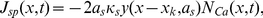
(31)where 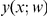 is a rectangle function of width 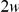:
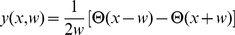
(32)and 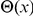 is the Heaviside step-function (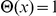 for 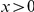 and zero otherwise). The rate, 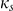, is given by the inverse of the mean first passage to reach a small opening of radius 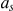. Thus
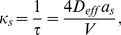
(33)where 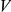 is the compartment volume. The total flux of calcium ions into the neck of 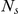 spines with a neck radius 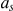 distributed at positions 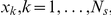 is given by
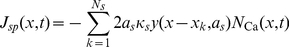
(34)and
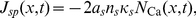
(35)where 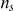 is the spine density per unit length.

#### Calcium dye

The effect of the calcium dye is modeled as buffer by the reaction
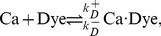
(36)where 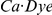 denotes the calcium-dye complex. The kinetic equations are given by
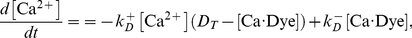
(37)

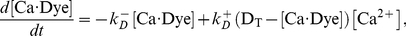
(38)where 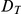 is the total dye concentration, i.e., 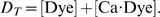


#### Synaptic input

Calcium influx on dendritic spines is mediated primarily by slow NMDA currents [56]. The voltage dependent NMDA channel is at resting potential mostly block by the 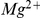 ions. As [57], we ignore the details of the voltage dependence of the NMDA receptor channel and consider a simplified model corresponding to presynaptic stimulation in conjunction with postsynaptic voltage clamp. The time course of the NMDA mediated synaptic current is modeled as the difference of two exponentials
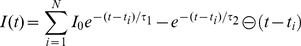
(39)where 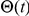 denotes the step-function, 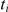 is the time of stimulus initiation and 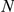 the number of pulses. The electrical currents are transformed into a particle current per unit length according to
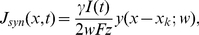
(40)where 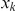 is the location of the receptor, 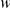 is the radius of the channel opening, 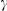 the fraction of current carried by calcium through the receptor, 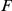 the Faraday constant, 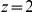 the valence of calcium and 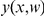 is a rectangular function with center at 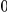 and half-width 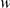.

#### Reaction-diffusion equations

The total effect of buffers, pumps and spines on the cytosolic calcium concentration can be summarized in form of a reaction-diffusion equation:
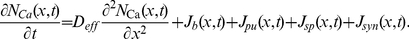
(41)where 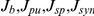 describes the calcium fluxes due to the buffers, pumps, spines and synaptic input. Equation (41) is coupled to the dynamic equations for the particle density of 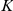 buffer molecules 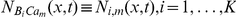 with maximally 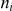 calcium binding sites and to the equation describing the calcium-dye particle density, 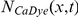. We finally obtain the set of equations that describe the calcium dynamics in the dendrite:
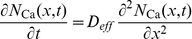
(42)

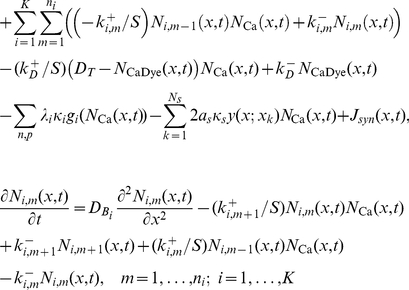
(43)

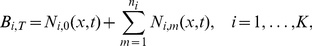
(44)

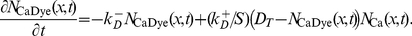
(45)


We included in the above equations the effect of mobile buffers. However, in the following, we assume that the buffers are fixed and set the buffer diffusion constants, 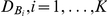, to zero.

#### Numerical simulations

The reaction-diffusion equations (42)–(45) were solved numerically using MATLAB. The partial differential equations were solved using the numerical method of lines which is implemented in the MATLAB solver. Space and time discretizations were set to 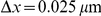 and 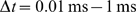, respectively, depending on the total simulation time which varied between 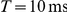 and 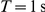. The total simulation time was determined by the biological components included in the simulation protocols. For example, for a simulation of calcium diffusion with activated pumps and buffers, a simulation time of 10 ms was sufficient due to the fast uptake of calcium by the buffers. Simulation protocols that included synaptic input required a much larger simulation time of about 1 s (Figure 4).

## Supporting Information

Video S1Movie of a stochastic simulation of 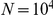 particles in a model dendrite with an attached spine. The geometric measurements for the dendritic segment and the dendritic spine were extracted from Figure 2A. The top and the bottom of the dendritic cylinder are absorbing surfaces.(AVI)

Video S2Movie of a diffusion experiment of 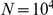 particles in a cylindrical domain (radius 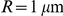, length 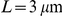) with absorbing boundary conditions at the top and bottom of the cylinder. Diffusion constant: 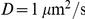.(AVI)

Text S1 Validation study for testing the algorithm implemented in the stochastic simulation tool.
(PDF)

Figure S1(A) Global particle concentration 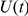 in a cylindrical domain (radius 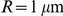, length 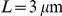) with absorbing top and bottom and normalized local particle concentration 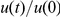 in a small sampling volume with center at 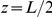 and height 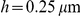. Comparison of the exact global and local particle concentrations (7) and (8), respectively, to the Brownian simulation results using 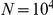 particles. (B) Comparison of the averaged mean first passage time as a function of cylinder length 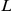. Diffusion constant: 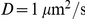.(EPS)
