# Supplementary material for: Barriers to Diffusion in Dendrites and Estimation of Calcium Spread Following Synaptic Inputs
Source: PLoS Comput Biol. 2011 Oct 13;7(10):e1002182. doi: 10.1371/journal.pcbi.1002182 (PMC3192802; doi:10.1371/journal.pcbi.1002182)
Supplement: Text S1 — Validation study for testing the algorithm implemented in the stochastic simulation tool. (PDF) [file pcbi.1002182.s003.pdf]

Text S1

We tested the correctness of the algorithm implemented in the Monte Carlo simulation tool for a simple diffusion experiment in a cylindrical domain (radius $R = 1\,\mu$m, length $L = 3\,\mu$m) with absorbing boundary conditions at the top and bottom of the cylinder. For this geometry the spatiotemporal development of the particle distribution can be given in analytical form. We compared three measures with the simulation results: (i) the global particle concentration in the cylindrical domain, (ii) the local particle concentration measured in a small cylindrical sampling volume, (iii) the MFPT to the absorbing boundaries.
The particle concentration u satisfies the following set of equations

$$\frac{\partial u(z,t)}{\partial t} = D\,\frac{\partial^2 u(z,t)}{\partial z^2}\,, \tag{1}$$

$$u(z,0) = u_0(z)\,, \tag{2}$$

$$u(z=0,t) = 0\,, \tag{3}$$

$$u(z=L,t) = 0\,, \tag{4}$$

Separation of variables leads to the solution

$$u(z,t) = \sum_{n=1}^{\infty} A_n \exp\Big[-\big(\frac{\pi n}{L}\big)^2 Dt\Big] \sin\frac{n\pi z}{L}\,, \tag{5}$$

with

$$A_n = \frac{2}{L}\int_0^L u_0(z)\sin\frac{n\pi z}{L}\,dz\,. \tag{6}$$

For the global concentration we obtain for a uniformal initial distribution

$$U(t) = \frac{1}{|\Omega|}\int_\Omega u(\boldsymbol{x},t)d\boldsymbol{x} = \frac{1}{L}\int_0^L u(z,t)dz = \sum_{n=1}^{\infty} 8\exp\Big[-\big(\frac{\pi n}{L}\big)^2 Dt\Big]\frac{\sin^4(\frac{n\pi}{2})}{n^2\pi^2}\,, \tag{7}$$

where $|\Omega| = \pi R^2 L$ is the volume of the cylinder.
Similarly, for a cylindrical sampling volume of height h with center at $z = L/2$ and a uniform initial distribution ($u_0(z) = 1/L$) we get for the local concentration in the sampling volume

$$u(t) = \frac{1}{L}\int_{L/2-h/2}^{L/2+h/2} u(z,t)dz = \sum_{n=1}^{\infty} 8\exp\Big[-\big(\frac{\pi n}{L}\big)^2 Dt\Big]\frac{\sin^3(\frac{n\pi}{2})\sin(\frac{hn\pi}{2L})}{n^2\pi^2}\,. \tag{8}$$

The MFPT $\tau(z)$ to the absorbing boundary from a location $z\,(0 < z < L)$ is given by

$$D\,\frac{\partial^2\tau(z)}{\partial z^2} = -1\,,$$

$$\tau(z=0) = 0\,,$$

$$\tau(z=L) = 0\,,$$

which leads to $\tau(z) = z(L-z)/(2D)$. The averaged MFPT for a uniform initial distribution is

$$E\tau = \frac{\pi R^2}{|\Omega|}\int_0^L \frac{z(L-z)}{2D}dz = \frac{L^2}{12D}\,. \tag{9}$$

Brownian simulation in the cylindrical simulation domain were performed with $N = 10^4$ particles and $D = 1\,\mu\text{m}^2/\text{s}$. The comparison of the simulation results with the analytical expressions (7), (8) and (9) are shown in Figure 1. A good agreement between the simulations and the exact results was obtained, thus, providing strong evidence for the correctness of the algorithm implemented in the simulation tool. We remark that the size of the time-step dt was crucial for the correct determination of the MFPT time. Larger time steps than $dt = 10^{-5}\,\text{s}$ led to significant deviations from the exact result (**??**). A movie of the test simulation is included (Video S2).
